# Association of uric acid and red blood cell distribution width with severe obstructive sleep apnea–hypopnea syndrome in middle-aged males

**DOI:** 10.1371/journal.pone.0354161

**Published:** 2026-07-17

**Authors:** Tingting Zhou, Hongyan Tao, Hong Wang, Fengxian Wei

**Affiliations:** 1 Department of Respiratory and Critical Care Medicine, The Second Hospital of Lanzhou University, Lanzhou, Gansu, China; 2 The Second Hospital & Clinical Medical School, Lanzhou University, Lanzhou, Gansu, China; 3 Department of General Surgery, The Second Hospital of Lanzhou University, Lanzhou, Gansu, China; University of Diyala College of Medicine, IRAQ

## Abstract

**Background:**

Obstructive sleep apnea-hypopnea syndrome (OSAHS) is sleep-related breathing disorder with high prevalence. Polysomnography is the gold diagnosis standard. Routine clinical biomarkers would assist to reflect disease severity. Our study aimed to explore the association between hematological parameters and newly diagnosed severe OSAHS in middle-aged males.

**Methods:**

This retrospective study enrolled 132 cases (40 ~ 65 years). Patients were divided into non-severe group (n = 69) and severe group (n = 63) based on the apnea-hypopnea index (AHI). Hematological parameters were compared between the two groups. Unitarian and multivariate logistic regression were performed to identify factors independently correlated with severe OSAHS. Receiver operating characteristic (ROC) curve analysis was performed to evaluate discriminative performance. Spearman correlation and multiple linear regression were utilized to investigate correlated factors with sleep hypoxia.

**Results:**

Significantly higher hemoglobin, hematocrit (HCT), mean corpuscular volume (MCV), red blood cell distribution width-standard deviation (RDW-SD), and uric acid were found in the severe group than the non-severe group (all P < 0.05). Multivariate logistic regression identified uric acid as the only independent correlate of severe OSAHS (OR = 1.01, P = 0.012). The combination of uric acid and RDW-SD yielded an AUC of 0.79 (95% CI: 0.69–0.89), with a sensitivity of 77.4% and specificity of 80.4%. Spearman analysis showed that uric acid correlated significantly with sleep mean SpO_2_ (r = –0.26, P = 0.034) and sleep minimum SpO_2_ (r = –0.30, P = 0.014), but not with AHI (r = 0.19, P = 0.123). In multiple linear regression adjusted for age and BMI, both uric acid and RDW-SD remained independently associated with sleep mean and minimum SpO_2_ (P < 0.001).

**Conclusions:**

Uric acid and RDW-SD are independently associated with OSAHS severity and sleep hypoxia in middle-aged males. The combination of these two routine clinical markers may offer useful information for risk stratification, while prospective studies is needed for validation.

## Introduction

Obstructive sleep apnea-hypopnea syndrome (OSAHS) is one of the most common sleep-related breathing disorders. In China, it is estimated that approximately 176 million individuals aged 30–69 years suffer from OSAHS, with China currently ranking first globally [1]. OSAHS can cause daytime fatigue, somnolence, anxiety, and depression, and is a frequent cause of decreased work efficiency and traffic accidents [2]. More importantly, severe patients not only have an increased risk of nocturnal sudden death but also suffer from long-term multi-system damage to the cardiovascular, cerebrovascular, and endocrine systems, significantly increasing the risks of hypertension, coronary heart disease, stroke, insulin resistance, hyperuricemia, cognitive impairment, and dementia [3].

Polysomnography (PSG) is the gold standard for the diagnosis of OSAHS [4]. However, in non-specialized settings, limited access to PSG has led to the widespread use of screening questionnaires and portable monitors [4,5]. Nevertheless, these alternatives would be influenced by environmental and technical variability and show suboptimal diagnostic agreement with PSG, with missed diagnosis rates reported to be as high as 48.2% [6]. Therefore, there is continued interest in identifying simple, low-cost clinical biomarkers that may assist in risk stratification, particularly for identifying severe patients who are at the highest risk of complications.

Complete blood count and biochemical parameters are widely available, stable, and inexpensive in clinic, and previous studies have reported a close relationship between them and OSAHS [7]. Uric acid and red blood cell distribution width (RDW) are particularly associated with oxidative stress and inflammation,which are key pathways in OSAHS pathophysiology [3]. In OSAHS, hypoxia-induced impaired erythropoietic and shortened red blood cell survival may lead to elevated RDW, while intermittent hypoxia would activate xanthine oxidase, thereby increasing uric acid production [8–10]. However, few studies have systematically examined the combined value of uric acid and RDW for reflecting OSAHS severity in a specific population.

We therefore aimed to explore the association of uric acid and RDW with OSAHS severity in a cohort of middle-aged males. Given the cross-sectional design and the absence of a healthy control group, this analysis is exploratory and hypothesis-generating, and aims to evaluate whether uric acid and RDW could serve as adjunctive correlates of disease severity.

## Materials and methods

### Study population

According to the inclusion and exclusion criteria, middle-aged male patients with newly diagnosed OSAHS who were hospitalized at the Department of Respiratory and Critical Care Medicine, Lanzhou University Second Hospital, from January 2024 to December 2024 were enrolled as study subjects. Inclusion criteria were: (1) male; (2) age 40–65 years; (3) stable long-term residence, with no travel history exceeding 1 month within the year; (4) available hematological parameter test results; (5) PSG-diagnosed OSAHS, with no previous diagnosis or treatment history. Exclusion criteria were: (1) previous OSAHS treatment history; (2) surgical and anesthesia history within 3 months; (3) long-term medication history; (4) alcohol abuse or psychiatric disease history; (5) chronic cardiopulmonary, hepatic, renal, hematological, or autoimmune diseases; (6) recent infection history; (7) inability to cooperate or obtain valid information. According to the apnea-hypopnea index (AHI) in the guidelines (8), patients were divided into non-severe group (5 ≤ AHI ≤ 30 events/h) and severe group (AHI > 30 events/h).

We restricted our cohort to males aged 40–65 years to reduce confounding. OSAHS prevalence elevated in this age group and is substantially higher in males (male-to-female ratio ~ 2–3:1) [11]. Older adults have more comorbidities and age-related hematological changes that differ from middle-aged populations [12], and gender differences in clinical presentation, pathophysiology, and laboratory reference ranges [13–15] would introduce further heterogeneity. We also included only newly diagnosed, treatment-naive patients to better capture the baseline pathophysiological state.

This was a retrospective study. Patient information was protected throughout the study process, and data were accessed for research purposes on 04/02/2026 after ethical review and approval by the Ethics Committee of Lanzhou University Second Hospital (2026A-183), and the individual participant information was blinded and could not be identified.

### Polysomnography

All patients completed PSG examination during hospitalization. In a dedicated sleep room under quiet nighttime conditions, a polysomnography monitor (SOMNOscreen™ plus PSG+ system, SOMNOmedics, Germany) was used to monitor and record sleep video, snoring sounds, body position, AHI, respiratory airflow signals, thoracic and abdominal respiratory inductance plethysmography (for respiratory effort), oxygen saturation, electrocardiogram, electroencephalogram, electrooculogram, submental electromyogram, and lower limb electromyogram in real-time, with monitoring time ≥7 hours. The following day, automated preliminary scoring was done by using DOMINO software (version 3.0, SOMNOmedics, Germany), and with manual verification by an experienced technologist following AASM 2012 criteria [16].

### Hematological parameters detection and calculation

The analyzed hematological parameters included three aspects: complete blood count, rapid biochemical tests, and calculated parameters. After fasting venous blood collection in the early morning, samples were sent to the hospital laboratory center for detection utilizing automated analyzers, and reports were issued by professional laboratory personnel. Neutrophil-to-lymphocyte ratio (NLR) and platelet-to-lymphocyte ratio (PLR) were calculated according to reference [17].

### Statistical analysis

SPSS 22.0 software (IBM Corp., Armagh, NY, USA) was used for statistical analysis. All hematological parameters were numerical variables. Data with a normal distribution were expressed as mean ± standard deviation (x̄ ± s), and group comparison was performed using T test directly or after logarithmic transformation; otherwise, data were expressed as median and interrogative range [M (Q1, Q3)], and compared using the Mann–Whitney U test. Categorical variables were compared using the chi-square test.

Univariate logistic regression was performed to identify hematological parameters associated with severe OSAHS. Variables with P < 0.10 while without high collinearity were then entered into a multivariate logistic regression model to determine factors independently associated with severe OSAHS. Receiver operating characteristic (ROC) curves were generated to evaluate the discriminative ability of parameters for distinguishing severe from non-severe OSAHS patients, and the optimal cut-off values were determined using the You-den index. Spearman correlation analysis was used to examine the relationships between hematological parameters (uric acid and RDW-SD) and hypoxia indices (AHI, mean SpO_2_, and minimum SpO_2_). Subsequently, multiple linear regression models were performed with mean SpO_2_ and minimum SpO_2_ as separate dependent variables, adjusting for age and BMI, to determine whether the associations were independent of potential confounders. P value < 0.05 was considered statistically significant.

## Results

### General characteristics

A total of 132 middle-aged male patients with newly diagnosed OSAHS were enrolled, with mean age of 50.69 ± 6.28 years and mean body mass index (BMI) of 28.09 ± 3.71 kg/m². Based on AHI severity, 69 cases (52.3%) were divided into non-severe group and 63 cases (47.7%) were severe group. No significant differences were observed in age, BMI, or comorbidities between the two groups (P > 0.05). Significant differences were found in AHI, sleep mean SpO_2_, and sleep minimum SpO_2_ (P < 0.05) (Table 1).

**Table 1:** General characteristics of enrolled patients.

Characteristic	Non-severe (n = 69)	Severe (n = 63)	T/χ²	P
Age (years)	50.88 ± 6.38	50.10 ± 6.32	0.63	0.532
BMI (kg/m²)	27.62 ± 3.14	28.90 ± 4.58	−1.57	0.121
AHI (events/h)	15.74 ± 7.49	46.41 ± 12.05	−13.36	<0.001
Mean SpO_2_ (%)	93 (92, 95)	89 (84.75, 92)	−5.16	<0.001
Minimum SpO_2_ (%)	77.85 ± 9.15	65.00 ± 8.35	6.63	<0.001
Smoking history (n, %)	20 (29.0)	18 (28.6)	0.003	0.958
Alcohol consumption (n, %)	11 (15.9)	14 (22.2)	0.85	0.358
Hypertension (n, %)	8 (11.6)	13 (20.6)	2.01	0.156
Diabetes mellitus (n, %)	5 (7.2)	7 (11.1)	0.60	0.441

### Comparison of hematological parameters between groups

Patients with severe OSAHS exhibited significantly higher levels of hemoglobin (HGB), hematocrit (HCT), mean corpuscular volume (MCV), red blood cell distribution width-standard deviation (RDW-SD), and uric acid than patients in the non-severe group (P < 0.01). No statistically significant differences were found in the other parameters such as neutrophil count, lymphocyte count, monocyte count, red blood cell distribution width-coefficient variation (RDW-CV), platelet parameters, lipid profile, immature granulocyte percentage (IG%), or NLR between the two groups (Table 2).

**Table 2:** Comparison of hematological parameters between groups.

Variable	Non-severe (n = 69)	Severe (n = 63)	T/Z	P
Neutrophils (×10^9^/L)	4.07 ± 0.98	4.17 ± 1.32	−0.43	0.669
Lymphocytes (×10^9^/L)	1.84 ± 0.512	1.77 ± 0.63	0.61	0.545
Monocytes (×10^9^/L)	0.48 ± 0.16	0.50 ± 0.15	−0.63	0.530
HGB (g/L)	165.16 ± 17.59	176.79 ± 23.75	−2.51	0.015
HCT (L/L)	0.49 ± 0.06	0.54 ± 0.08	−2.83	0.007
MCV (fL)	91.53 ± 4.64	94.38 ± 5.05	3.90	0.024
RDW-SD (fL)	42 (41, 45)	45 (43, 50.25)	−3.36	0.001
RDW-CV (%)	12.8 (12.1, 13.4)	12.9 (12.3, 14.65)	−1.48	0.139
Platelets (×10^9^/L)	202.11 ± 72.41	185.91 ± 69.19	1.07	0.288
PDW (fL)	14.77 ± 3.21	14.47 ± 3.58	0.42	0.679
MPV (fL)	11.35 ± 1.29	11.10 ± 1.32	0.88	0.383
PCT (%)	0.23 ± 0.07	0.21 ± 0.058	1.44	0.154
IG (×10^9^/L)	0.2 (0.1, 0.3)	0.02 (0.02, 0.03)	−0.47	0.640
IG%	0.36 ± 0.20	0.35 ± 0.15	0.07	0.947
Total cholesterol (mmol/L)	1.80 (1.26, 2.80)	1.87 (1.31, 2.57)	−0.04	0.965
Triglycerides (mmol/L)	3.90 ± 0.93	4.20 ± 0.99	−1.47	0.145
HDL-C (mmol/L)	1.03 ± 0.25	0.97 ± 0.24	1.18	0.241
LDL-C (mmol/L)	2.57 ± 0.85	2.82 ± 0.99	−1.26	0.212
Uric acid (μmol/L)	392.28 ± 96.35	441.61 ± 91.98	−2.38	0.020
NLR	2.19 (1.75, 2.71)	2.37 (1.80, 2.96)	−0.84	0.403
PLR	115.12±39.63	111.40±46.29	0.423	0.674

### Factors associated with severe OSAHS

Univariate Logistic analysis was performed with severe OSAHS as the dependent variable. HGB, HCT, MCV, RDW-SD, IG%, and uric acid were found to be statistically associated with severe OSAHS (P < 0.05) (Table 3).

**Table 3:** Univariate analysis of severe OSAHS.

Variable	B	SE	Wald χ²	P
Neutrophils (×10^9^/L)	0.08	0.19	0.19	0.666
Lymphocytes (×10^9^/L)	−0.24	0.39	0.37	0.541
Monocytes (×10^9^/L)	0.86	1.35	0.41	0.523
HGB (g/L)	0.03	0.01	6.51	0.011
HCT (L/L)	9.19	3.31	7.74	0.005
MCV (fL)	0.12	0.05	6.73	0.009
RDW-SD (fL)	5.95	1.94	9.46	0.002
RDW-CV (%)	0.14	0.09	2.15	0.143
Platelets (×10^9^/L)	−0.003	0.003	1.13	0.289
PDW (fL)	−0.28	0.07	0.18	0.675
MPV (fL)	−0.16	0.18	0.77	0.379
PCT (%)	−5.55	3.92	2.01	0.157
IG (×10^9^/L)	−1.14	18.24	0.004	0.949
IG%	0.78	0.29	6.96	0.008
Total cholesterol (mmol/L)	0.33	0.23	2.11	0.147
Triglycerides (mmol/L)	0.11	0.16	0.43	0.514
HDL-C (mmol/L)	−1.07	0.91	1.38	0.239
LDL-C (mmol/L)	0.29	0.24	1.55	0.213
Uric acid (μmol/L)	0.005	0.002	5.06	0.024
NLR	0.26	0.19	1.86	0.173
PLR	−0.002	0.005	0.18	0.670

Multivariate logistic regression analysis was performed with severe OSAHS as the dependent variable, including MCV, RDW-SD, HCT, and uric acid as autonomous variables. After adjustment, uric acid remained the only independent factor significantly associated with severe OSAHS (OR = 1.01, 95% CI: 1.00–1.01, P = 0.012). While MCV (P = 0.139), RDW-SD (P = 0.322), and HCT (P = 0.435) did not achieve statistical significance (Table 4).

**Table 4:** Multivariate Analysis of Severe OSAHS.

Variable	B	P	OR	95% CI
RDW-SD (fL)	0.06	0.322	1.06	0.95–1.18
HCT (%)	0.04	0.435	1.04	0.95–1.13
MCV (fL)	0.09	0.139	1.10	0.97–1.25
Uric acid (μmol/L)	0.01	0.012	1.01	1.00–1.01

### Exploratory discriminative ability of uric acid and RDW-SD

ROC curve analysis was carried out to explore the ability of uric acid and RDW-SD to discriminate between severe and non-severe OSAHS patients. For uric acid alone, the AUC was 0.70 (95% CI: 0.58–0.81) at a cut-off of 392.5 umol/L, with a sensitivity of 77.4% and a specificity of 60.7%.

When combined with RDW-SD (cut off: 42.5 fL), the discriminative performance improved modestly, yielding an AUC of 0.79 (95% CI: 0.69–0.89), with a sensitivity of 77.4% and a specificity of 80.4%. Combinations with HCT, MCV, and HGB also improved the AUC compared with uric acid alone, though the UA + RDW-SD combination showed the highest AUC among the tested pairs (Table 5).

**Table 5:** ROC analysis of uric acid combined with different parameters.

Parameter	AUC (95% CI)	Cut-off	Sensitivity (%)	Specificity (%)
UA	0.70 (0.58–0.81)	392.5	77.4	60.7
UA + RDW-SD	0.79 (0.69–0.89)	42.5	77.4	80.4
UA + HCT	0.77 (0.66–0.87)	0.523	45.2	85.1
UA + MCV	0.75 (0.64–0.86)	94.35	58.1	78.6
UA + HGB	0.75 (0.69–0.89)	182.5	41.9	85.7

### Correlates of sleep hypoxia indices

To investigate the associations between hematological parameters and sleep hypoxia indices, Spearman correlation analyses were first performed. RDW-SD showed significant positive correlation with AHI (r = 0.34, P = 0.002) and significant negative correlations with both sleep mean SpO_2_ (r = −0.50, P < 0.001) and sleep minimum SpO_2_ (r = −0.43, P < 0.001). In contrast, uric acid was substantially correlated with sleep mean SpO_2_ (r = −0.26, P = 0.034) and sleep minimum SpO_2_ (r = −0.30, P = 0.014), but not with AHI (r = 0.19, P = 0.123). This differential pattern suggested that uric acid may be more specifically related to the degree of nocturnal hypoxia than the frequency of apneic events.

Multiple linear regression models were then performed for sleep mean and minimum SpO_2_, adjusting for age and BMI (Table 6). Both RDW-SD and uric acid remained independently associated with both sleep hypoxia indices.

**Table 6:** Multiple linear regression models for nocturnal hypoxia indices.

Dependent variable	Independent variable	B (95% CI)	Standardized β	P
Mean SpO₂ (%)	RDW-SD	–0.43 (–0.56, –0.30)	–0.61	<0.001
Minimum SpO₂ (%)	RDW-SD	–0.63 (–0.95, –0.32)	–0.44	<0.001
Mean SpO₂ (%)	Uric acid	–0.02 (–0.03, –0.004)	–0.32	0.011
Minimum SpO₂ (%)	Uric acid	–0.03 (–0.06, –0.008)	–0.32	0.011

*All models were adjusted for age and BMI.

## Discussion

Severe OSAHS conferred substantial risks of systematic complications. Although PSG remains the diagnostic gold standard, its limited availability has prompted interest in identifying simple and low-cost biomarkers that may reflect disease severity [7,9,18]. In this retrospective study of middle-aged males with newly diagnosed OSAHS, uric acid and RDW-SD were investigated to be significantly elevated in severe compared with non-severe group. And uric acid was independently associated with severe OSAHS after adjustment for potential confounders. Furthermore, both markers showed significant independent correlations with sleep hypoxia indices, suggesting their potential utility as adjunctive correlates of disease severity.

The clinical features of OSAHS include recurrent upper airway collapse, apnea, and chronic intermittent hypoxia, which would activate xanthine oxidase, a key enzyme in purine metabolism, thereby increasing uric acid production [19]. In addition, hypoxia-induced oxidative stress may reduce renal uric acid excretion through renal tubular injury, vasoconstriction-mediated decreases in renal blood flow, and competitive inhibition by lactate generated via anaerobic metabolism [20,21]. Similarly, hypoxia-driven erythropoietic changes, particularly those affecting red blood cell count, size distribution, and morphology, are consistent with the finding of elevated RDW-SD observed in severe OSAHS. Under chronic hypoxia conditions, increased erythropoietin (EPO) secretion stimulates compensatory erythrocyte proliferation, while concurrent inflammatory stress may lead to the release of immature erythrocytes of heterogeneous size, manifesting as elevated RDW-SD [10, 18]. In the current study, HGB, HCT, and MCV were also elevated in severe OSAHS, which is consistent with previous reports [22,23].

However, RDW-SD, MCV and HCT were found to be not retained statistically significant in multivariate analyses, possibly owing to their collinearity and also due to their variance was partially captured by uric acid and RDW-SD. The combination of uric acid and RDW-SD yielded an AUC of 0.79 for discriminating severe from non-severe OSAHS patients in this cohort. Although this performance does not support their use as a community screening tool in place of validated questionnaires such as STOP-Bang [4,24,25], the high specificity (80.4%) of the combined model may offer adjunctive information in settings where routine laboratory tests are already completed.

Our correlation analysis further revealed that RDW-SD was significantly correlated with both hypoxia indices and AHI, whereas uric acid was significantly correlated with both hypoxia indices but not with AHI. This suggested that UA elevation would be more specifically driven by the depth of hypoxia rather than the frequency of apneic events. In multiple linear regression models adjusting for age and BMI, both UA and RDW-SD remained independently associated with mean and minimum SpO_2_, with RDW-SD showing larger effect sizes than uric acid. Although the correlation between AHI and hypoxia indices is well documented [26], these parameters capture different dimensions of OSAHS severity—AHI frequency versus physiological impact of hypoxia [27,28].

Certain inflammatory markers in current study, including neutrophil-to-lymphocyte ratio (NLR) and immature granulocyte percentage (IG%), were found to be not differ substantially between severe and non-severe groups. While these markers have shown value in other disease contexts [29,30], their lack of association in our cohort were mainly considered to be due to the relatively modest sample size. It remains possible that chronic inflammatory pathways contributed to OSAHS-related morbidity [10]; however, our data suggested that hypoxia-driven erythrocyte and metabolic changes may be more directly related to disease severity in middle-aged males.

Several limitations should be mentioned. First, this was a single-center study with a modest sample size (n = 132) in Gansu province in China (average altitude: 1000–1500 meters). Second, the study was restricted to middle-aged males (40–65 years). While this design enhances internal validity, our findings may not apply to women, older adults, or other ethnic groups. Third, we lacked data on renal function, medications, and dietary factors [31,32], which may influence uric acid levels and for which we could not fully adjust. Fourth, we used a single PSG recording per patient and did not account for night-to-night variability. Fifth, the absence of a healthy control group (AHI < 5) means that our ROC analysis reflects discrimination between severe and non-severe diagnosed OSAHS patients, not screening performance in the general community. Finally, the lack of external validation of our predictive model precludes its immediate clinical application. Despite these limitations, our study provides hypothesis-generating evidence that uric acid and RDW-SD are independently associated with OSAHS severity and nocturnal hypoxia in middle-aged males. If confirmed by large scale studies, these simple, widely available biomarkers may serve as adjunctive tools for risk stratification and may inform future investigations into treatment monitoring and mechanistic pathways.

## Conclusion

Uric acid and RDW-SD were significantly associated with the severity and the degree of sleep hypoxia in middle-aged males of newly diagnosed OSAHS. These available parameters may offer adjunctive information for risk stratification. Prospective validation in large-scale studies is warranted before any clinical application can be recommended.
